# Falls and fear of falling in older adults with total joint arthroplasty: a scoping review

**DOI:** 10.1186/s12891-019-2954-9

**Published:** 2019-12-12

**Authors:** Serena Kuangyi Chen, Don Voaklander, Danielle Perry, C. Allyson Jones

**Affiliations:** 1 School of Public Health, University of Alberta Edmonton, Alberta, T6G 1C9 Canada; 2 Department of Physical Therapy, University of Alberta Edmonton, Alberta, T6G 2G4 Canada

**Keywords:** Total joint arthroplasty, Falls, Fear of falling, Older adults, Osteoarthritis

## Abstract

**Background:**

Patients waiting or recovering from total joint arthroplasty (TJA) are at risk for falls which can lead to restriction of activity and negatively impact recovery. The objective of this scoping review is to critically appraise and synthesize the evidence in the reported number of falls, fear of falling, and risk factors associated with falls in older patients waiting for or recovering from TJA.

**Methods:**

Seven electronic databases were searched with no date limits and using language restriction (English). The inclusion criteria were 1) cohorts that included older adults 60+ years of age, 2) reported prevalence of falls, fear of falling, and/or risk factors for falls in patients who were waiting or recovering from TJA and 3) cross-sectional studies, cohort studies, and case control study designs. The quality assessment of selected articles was assessed using the SIGN Guidelines Checklist.

**Results:**

Of the 866 citations identified, 12 studies met the inclusion criteria and were reviewed. Prevalence of falls in pre-operative TJA patients and post-operative TJA patients ranged from 23 to 63%, and 13 to 42%, respectively. Of those five studies that examined fear of falling, pre-operative TJA patients reported greater fear of falling than post-operative patients. Modifiable risk factors for falls included fear of falling, joint range of motion, and depression.

**Conclusions:**

An increased risk of falls in patients with TJA was reported both for patients waiting for and recovering from surgery. A number of modifiable risk factors were identified including fear of falling that could be targeted in fall prevention programs for TJA.

## Background

Falls in older adults is a major public health problem given that it is the leading cause of injury in community dwelling older adults [1]. Osteoarthritis (OA) which is a prevalent disabling condition in older adults, is associated with increased fall risk (OR: 2.4; 95% CI: 1.6, 5.4) [2]. While total joint arthroplasty (TJA) is an effective surgical option for advanced OA of the hip and knee [3], little evidence has examined falls in patients with TJA. The few reviews examining falls with TJA have reported a higher rate of falls with TKA and identified intrinsic risk factors such as balance and postural control [4, 5].

Causes of falls are typically multifaceted and can be attributed to intrinsic or extrinsic risk factors [6, 7]. Intrinsic risk factors for falls commonly seen in OA, include muscle weakness [8], balance deficits [9], pain [9], and fear of falling [7]. Others, however, have reported that balance deficits may not have a direct effect on falls in older adults with hip OA [10], yet balance deficits, knee muscle weakness, and increasing number of symptomatic joints were regarded as risk factors for falls with knee OA [11]. Pain is associated with muscle weakness, impaired balance and mobility limitations which may, in turn, be related to falls [12]. Evidence from a large longitudinal study reported that the risk of falls was greater for older women with lower extremity musculoskeletal pain; however, the risk was lowered with the use of analgesic medication [13]. Others have not found the use of analgesics for OA was associated with falls [9, 11].

Fear of falling is a modifiable risk factor for falls which limits mobility for older adults [14, 15]. Specifically, fear of falling is a constant concern about falling that can lead to self-imposed limitation of daily activities [16]. Fear of falling is a prevalent risk factor in older adults regardless of whether they have a history of falls. Anywhere up to 65% of older adults who have not fallen and 92% of those who have fallen have a fear of falling [17]. A high level of fear of falling after surgery can also reduce self-efficacy, or one’s perception of their ability. Although few studies have examined fear of falling in this patient population [4, 5], fear of falling is reported to be higher in patients with TJA as compared to community controls [18].

Because few systematic reviews have specifically defined or examined falls in TJA [4, 5, 19], the purpose of this scoping review is to critically appraise and synthesize the evidence for falls in patients with TJA and to map current evidence of falls, fear of falling and risk factors associated with falls in TJA patients during the perioperative period. The specific objective is to identify existing evidence on the reported number of falls, fear of falling, and known risk factors for falls in older adults who are waiting for or recovering from TJA.

## Methods

We conducted a scoping review using the method outlined by Arksey and O’Malley [20]. Inclusion criteria were 1) cohorts of older adults; 60+ years of age, 2) reported prevalence of falls, fear of falling, and/or risk factors for falls in patients who were waiting or recovering from TJA, and 3) cross-sectional studies, cohort studies, and case control study designs. Excluded studies consisted of articles that used indirect balance measures to predict falls such as Timed Up and Go, Dynamic Gait Index, and the Berg Balance Test.

### Data sources and search strategies

The search strategies were developed and implemented by a health sciences librarian for 7 electronic databases (Pubmed, Medline, CENTRAL, Embase, CINAHL Plus with Full Text, Web of Science, and SCOPUS) and was completed on April 6, 2017. The search strategy included the following keyword terms and concepts: 1) arthroplasty 2) knee joint or hip joint 3) replacement 4) osteoarthritis 5) accidental falls (Additional file 1: Appendix A). Research in-progress was searched via abstracts from Pubmed’s epub. Non-English studies with English abstracts were included in the literature search then excluded at the time of abstract review to provide an overview of possible publications in non-English journals. The decision to restrict to English articles was based on findings from systematic research evidence that reported no empirical evidence of bias was seen if papers written in languages other than English were excluded [21].

### Screening and data extraction

Citations were exported to Refworks and exact duplicates were electronically removed. The remaining citations were imported into COVIDENCE, a screening and data extraction tool, for abstract screening, full text review and data extraction [22]. Titles were reviewed for eligibility criteria by one reviewer (SC). Two reviewers (SC, DP) independently screened the remaining abstracts based on the inclusion criteria using a standard study selection form. Articles were then reviewed by two reviewers with “moderate” inter-rater reliability agreement (Kappa =0.69) [23]. Disagreement of article inclusion was resolved through consensus between reviewers or, if the reviewers did not arrive upon consensus, a third party adjudication (CAJ) was consulted for the final decision. One reviewer (SC) assessed the full-text articles. Data extraction was done by one reviewer (SC) and checked by a second reviewer (DP).

### Quality assessment

The SIGN Guidelines Checklist 3: Cohort Studies [24] was used to assess individual study quality through completion of a cohort or cross-sectional checklist. The checklist included 14 questions that addressed four areas: (1) selection of subjects (2) assessment of outcome, (3) confounding, and (4) statistical analysis. Studies were given either a “high”, “acceptable”, or “low” rating. Because of the methodological and clinical heterogeneity of the included studies, meta-analyses could not be conducted.

## Results

**Fig. 1:**
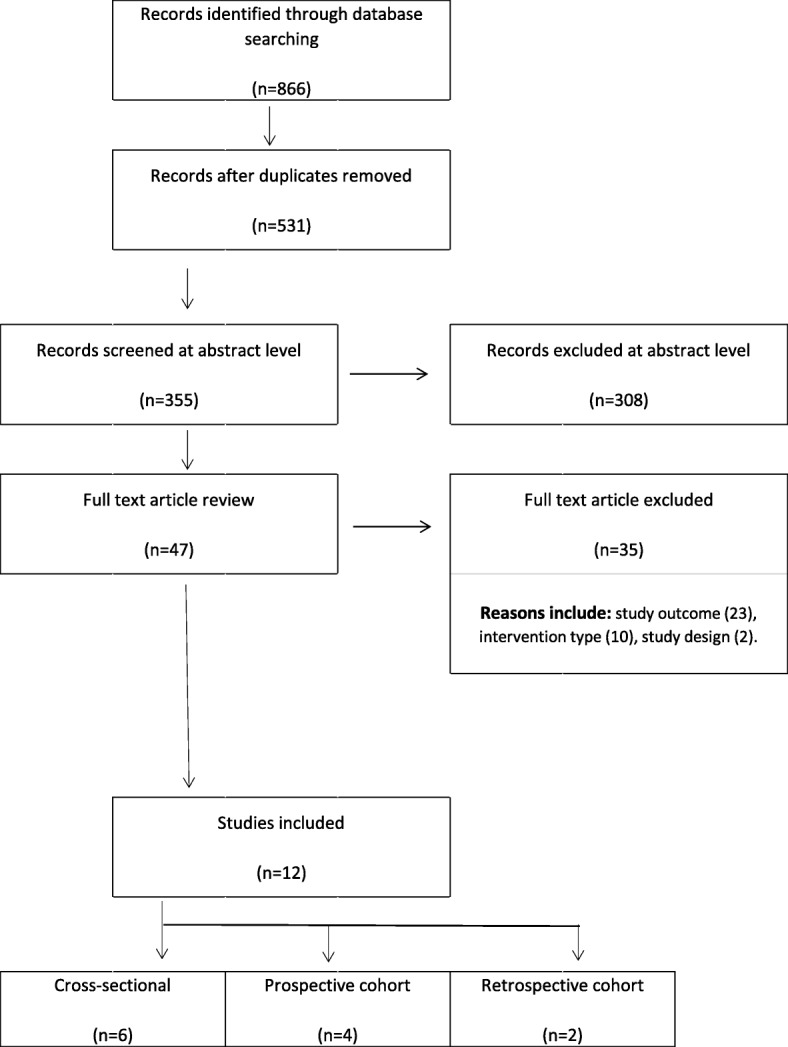
PRISMA Diagram

A total of 866 citations were retrieved: 293 in EMBASE with an additional 240 in SCOPUS, 174 in MEDLINE, 82 in Web of Science, 45 in CINAHL, 25 in CENTRAL, five in PubMed (of pub ahead of print only), two in Cochrane Central Register of Controlled Trials. All articles were imported into Covidence where 335 duplicates were removed. We assessed 47 full articles, of which 12 studies were included in this review [18, 25–35] regardless of methodological quality. The summary of study selection and reasons for exclusion is presented within the Preferred Reporting Items for Systematic Reviews and Meta-Analysis (PRISMA) diagram (Fig. 1). Two studies used the same cohort; however, had different primary objectives [30, 32]. Six studies were cross-sectional studies [25–30] of which one was an abstract [27], four were prospective cohort studies [18, 31–33], and two were retrospective cohort studies [34, 35] (Additional file 2: Table S1). Eight studies [25–29, 32, 33, 35] had a comparison group, comparing self-reported “fallers” to “non-fallers”.

The definition of falls varied among the studies. Six studies used a traditionally accepted definition of falls [25, 26, 30–33], which was “an unexpected event in which the participant comes to rest on the ground, floor, or lower level, not as a result of a major intrinsic event such as a faint or stroke, seizure, or an overwhelming external hazard”. The remaining studies did not explicitly define what constituted a fall in their study population [18, 27–29, 34, 35]. Sample sizes ranged from 31 to 413 participants. Data from the Osteoarthritis Initiative database were used for 2 studies with 413 [35] and 269 [34] participants. Three studies were conducted in the UK [28, 33, 34], two in Japan [25, 31], two in Australia [18, 29], two in USA [27, 35], two in Greece [30, 32] and one in Thailand [26].

Based on SIGN guidelines, four studies were poor quality [25–28] six were acceptable quality [18, 29, 30, 32, 34, 35] and two were high quality [31, 33]. Assignment of poor quality was due to several factors including missing confidence interval estimates (*n* = 1) and failure to discuss presence of potential bias (*n* = 2).

The mean ages of participants reported ranged from 63.9 ± 6.8 [32] to 75.0 ± 6.0 [31] years with cohorts comprised of 41% [34] to 100% [27] females. Seven studies examined total knee arthroplasties (TKA) [18, 26, 30–33, 35]; one pre-operative [30] and two post-operative [26, 31] and four both pre-operative and post-operative [18, 32, 33, 35]. Two articles examined total hip arthroplasties (THA) [25, 27], one preoperative [27] and the other post-operative [25]. Three studies examined TJA combining findings from both THA and TKA [28, 29, 34].

**Fig. 2:**
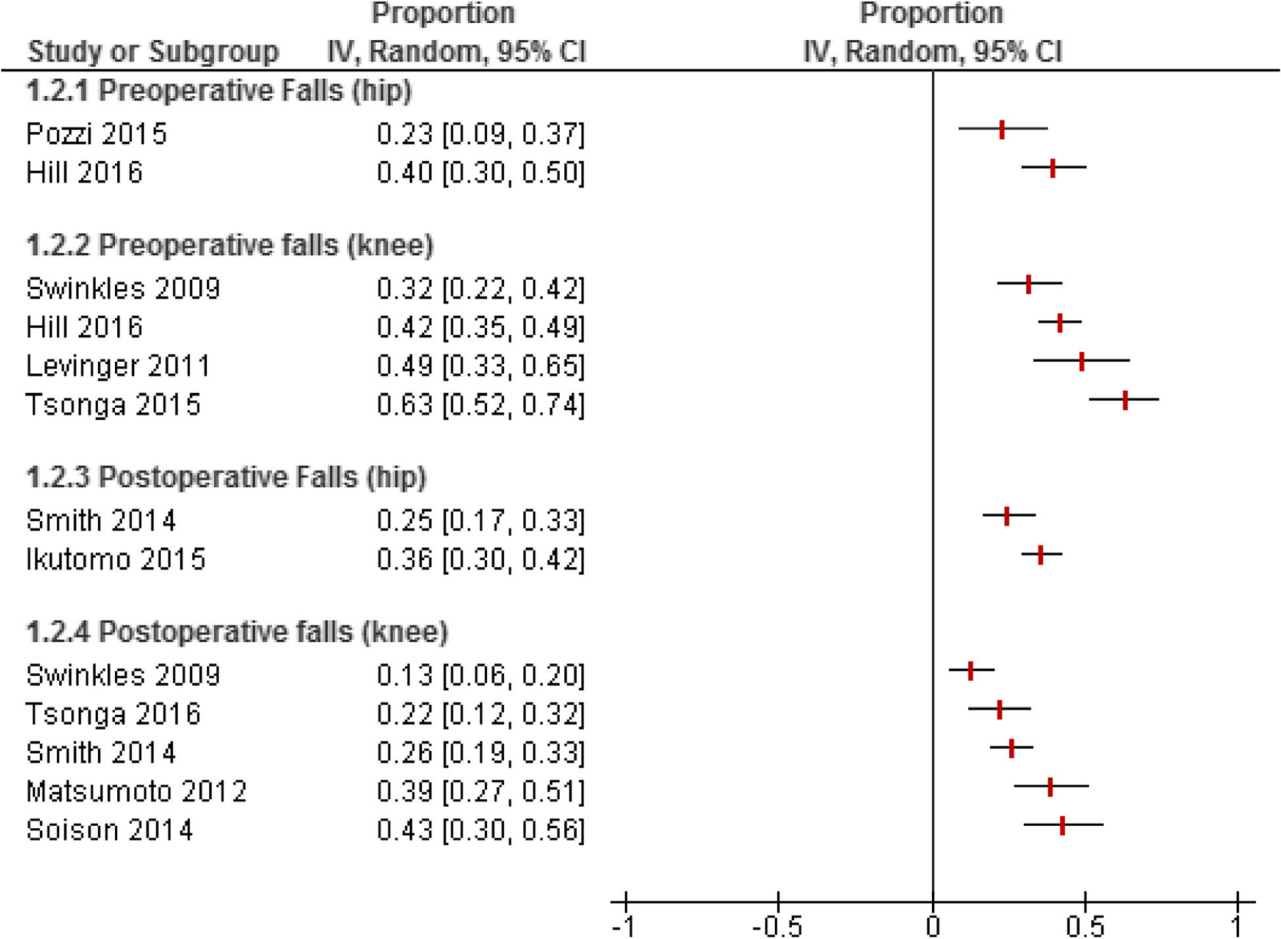
Proportion of pre and postoperative falls by type of joint replacement

The number of falls in three prospective cohort studies [18, 32, 33] reported a higher proportion of falls in pre-operative patients than during the post-operative recovery (Fig. 2). Of the 8 studies that examined pre-operative falls, the recall period ranged from one month to one year (Table 1). The number of reported falls ranged from 23% [27] to 63% [30]. Post-operative falls were reported in 7 articles and the number of falls ranged from 13% [33] to 42% [26]. Patients who were in the recovery phase were typically asked to recall falls over a one year period; however, post-operative recruitment ranged from 6 months to 6 years. One post-operative TKA study had a recall period that extended into the pre-operative phase and reported the greatest number of reported falls (42%) with a 6 year post-operative follow-up period [26].

**Table 1:** Number of reported falls before and after total joint arthroplasty

Author (year)	*n*	Joint arthroplasty	Recall time of falls				
Pre-operative Falls				Time before surgery		Number of falls	
Hill et al. (2016)	282	Hip (*n* = 85) Knee (*n* = 197)	12 Months	2–4 Weeks		116 (41%)	
Mitchell et al.(2007)	199	Hip (*n* = 84) Knee (*n* = 115)	1 Month	N.S.		75 (39%)	
Pozzi et al. (2015)	31	Hip	6 Months	2 Weeks		7 (23%)	
Tsonga et al. (2015)	68	Knee	12 Months	1 Month		43 (63%)	
Post-operative Falls				Time after surgery		Number of falls	
Smith et al. (2014)	269	Hip (*n* = 104) Knee (*n* = 165)	12 Months	12–72 Months		43 (26%)	
Ikutomo et al. (2015)	214	Hip	12 Months	12 Months		77 (36%)	
Matsumoto et al. (2012)	74	Knee	1 Month	6–11 Months		23 (39%)	
Soison et al. (2014)	54	Knee	12 Months	7–73 Months		23 (42%)	
Pre- & post-operative Falls				Time before surgery	Number of falls before TKA	Time after surgery	Number of falls after TKA
Levinger et al. (2011)	62	Knee (*n* = 35)	12 Months	N/S	17 (48%)	4 months	N/S
		Controls (*n* = 27)			8 (30%)		
Riddle et al. (2016)	413	Knee	12 Months	N/S	N/S	12 months–48 months	N/S
Swinkles et al. (2009)	99	Knee	3 Months (Pre) 12 Months (Post)	N/S	24/75 (32%)	1–12 months	11/85 (13%)
Tsonga et al. (2016)	68	Knee	1 Month	2 weeks	43 (63%)	1–12 months	15 (21%)

Abbreviations

*N/S* not stated, *TJA* total joint arthroplasty

### Fear of falling

Fear of falling was reported in five studies [18, 29–32] and used several different instruments including Activities-Specific Balance scale [36], Falls Efficacy Scale International (FES-I) [37] and the Modified Falls Efficacy Scale [38]. Fall status did not significantly affect fear of falling scores. Three of the five studies reported that fear of falling did not differ between fallers and non-fallers regardless of TJA pre- post-operative status [29, 31, 33]. One study reported that pre-operative patients were more fearful than those recovering from TJA surgery regardless of fall status (*p* < 0.001) [32] (Table 2). When compared to community dwelling older adults, patients with TKA had higher FES-I scores which was indicative of greater fear of falling (*p* < 0.01) [18].

**Table 2:** Fear of falling summary

Author	n	Measure	Comparison		*p*-value
			Faller	Non-Faller	
Hill et al. (2016)	282	ABC	63.8 ± 20.6	71.2 ± 21.1	0.051
Swinkles et al. (2009)	99	ABC	53.1 ± 26.4	64.4 ± 24.3	0.68
Matsumoto et al. (2012)	74	M-FES	122 ± 27.1	123 ± 23.3	0.87
			TJA cohort	Community cohort	
Levinger et al. (2011)	62	FES-I	11.4 ± 3.0	9.7 ± 2.9	< 0.01
			Pre-Operative	Post-Operative	
Tsonga et al. (2016)	68	ABC	63.7 ± 20.14	81.4 ± 16.2	< 0.001

Abbreviations

*ABC* Activities-Specific Balance scale, *FES-I* Falls Efficacy Scale – International, *M-FES* Modified Falls Efficacy scale, TJA total joint arthroplasty

### Risk factors for falls

#### Type of total joint arthroplasty (TKA/THA)

In one study that examined risk factors for falls in both THA and TKA patients, patients with TKA had a greater number of factors associated with falls than patients with THA [29]. Fear of falling was a risk factor in both joint groups, while pain, function and mental health were other risk factors reported in two TKA cohorts [29, 34].

#### Joint specific pain, stiffness and function

Of the twelve reviewed articles, seven articles measured joint pain, function and stiffness using the Western Ontario and McMaster Universities Osteoarthritis Index (WOMAC) scores [18, 26, 28–30, 32, 33]. Most studies did not find significant WOMAC differences between fallers and non-fallers after TJA. Two of the three studies that reported significant differences in WOMAC subscales: pain, stiffness, and function between faller and non-fallers were rated “poor” quality which may be indicative of confounding effects such as age [26, 28]. One cross-sectional study reported that 40% (*n* = 282) of pre-operative patients indicated that their fall was related to the painful joint [29].

#### Joint range of motion

Two studies which examined joint range of motion (ROM) and the risk of falls, reported significant joint range differences between fallers and non-fallers [27, 31]. Patients with TKA who had a 10-degree increase in knee flexion reduced the odds of falling by 72.3% (OR: 0.28; 95% CI: 0.09, 0.87) at 1-year after surgery; whereas, a 5-degree increase in ankle plantar flexion reduced the odds of falling by 40% [31]. In a cross-sectional study of 31 THA patients, total hip ROM and knee extension were significantly more restricted in fallers than non-fallers [27].

#### Number of comorbid conditions

The association between the number of comorbid conditions and falls is not clearly delineated in the TJA literature. Three of five studies that specifically examined comorbid conditions and falls, reported no differences in number of self-reported co-morbid conditions between fallers and non-fallers before [30, 33] and after TKA [32].

#### Depression

Of the five cross-sectional studies that examined falls and depression, three [28, 29, 35] reported a significant association between depression and a greater number of falls.

#### Medications

One cross-sectional study of 199 TJA patients reported a higher proportion of those with history of falls (13/75; 17%) took antidepressants as compared to those who had not fallen (8/119; 7%) (*p* = 0.02) [28]. No statistical adjustments for other factors were done. In contrast, a high quality longitudinal study concluded that use of antidepressants did not increase the risk of falls after adjusting for age, gender, number of comorbid conditions and pre-operative WOMAC scores (OR: 1.18, 95% CI: 0.16, 0.88) [33]. The use of other medications that may impact falls risk were not evaluated in any of the other studies.

## Discussion

Twelve articles were included in this scoping review that specifically examined falls in patients with TJA. Falls occurring during the peri-operative period of TJA is an emerging area of interest, with 10 of the 12 studies published after 2010. A preponderance of articles, however, were small, single-center studies with the majority examining TKA. Although 3 studies examined TJA as a single cohort, the type of joint may impact the risk of falls. The few studies that followed patients before and after surgery, reported a higher proportion of falls before surgery than afterwards. Risk factors, such as environmental hazards and use of walking assistive devices are known extrinsic risk factors for community-dwelling older adults [7] and have direct applicability for TJA, yet this type of risk factor was reported in few studies.

The pattern of falls during the perioperative period is an understudied area which has clinical implications. Within this review, two studies reported the number of falls increased dramatically as the number of risk factors increase [39, 40]. Patients waiting for TJAs may be at a high risk for falling due to increased pain and disability, while patients are also at a high risk of falling immediately after surgery due to limited mobility [41]. Up to 17% of patients admitted for short-term hospitalization reported a fall while in the hospital [41], reporting a higher incidence of in-hospital falls with TKA than THA [42].

Pain is a risk factor for falls in older adults with knee and back pain [43, 44]. It is possible that high levels of pain before surgery may, in part, account for the high number of falls with TJA. Presence of knee and hip pain may potentially explain the increased risk of falls in pre-operative patients compared to post-operative patients. Surprisingly, joint-specific pain as measured by the WOMAC was not identified as a risk factor for falls.

Restricted knee and ankle ROM was associated with falls with TKA, in particular, knee extension and ankle plantar flexion were associated with falls, while total hip ROM and knee extension were associated with falls with THA. These findings support the current evidence in the literature, that significant restricted hip ROM (extension, internal rotation), and ankle dorsiflexion were seen in fallers as compared to non-fallers [45]. Given the association with falls, increasing or maintaining ROM may be one modifiable factor in preventing falls.

Fear of falling is a well known risk factor for falls in OA patients [12]. Although TJA provides significant pain relief and large improvements with function [3], fear of falling may be problematic for this patient population, particularly when encouraging physical activity during recovery. Fear of falling is one of the few modifiable risk factors for falls, therefore adding cognitive behavioural interventions aimed at reducing fear of falling may be beneficial to improve long term functional outcomes in TJA patients post surgery [46]. Significant improvements have been reported with fear of falling in older adults with a combined program of exercise and education [47, 48].

The primary aim of this review was to determine the scope of the literature that assessed falls, fear of falling, and risk factors for falls in older adults during the perioperative TJA period. Of the 12 articles identified, the majority of studies were of low or moderate quality. More than half of the studies included in this review had small sample sizes (*n* < 74), which may not be reflective of the fall rate in this patient population. Methodological considerations of falls centred on defining what constituted a fall and the recall period. All studies used retrospective self-report of falls, which could lead to under-reporting, especially if the recall time is longer than 12 months [49]. Monthly falls diaries have been cited as a validated method of collecting falls data [50]; however, only two studies used this method. With advancements of personal activity monitors, electronic monitoring of falls may limit the bias associated with recalling and recording falls.

A consideration with any scoping review is the inclusion of all studies regardless of methodological quality. In this case, we examined methods used for ascertaining falls and fear of falling both before and after surgery. This approach allowed us to look at the evidence over the peri-operative period to identify possible gaps in the literature. While others have done systematic reviews on falls only with TKA [4, 5] or during the post-operative phase [19], we performed a scoping review which took a broader view looking at both THA and TKA, as well as falls during the peri-operative period. Because the literature was sparse, a scoping review provided a scan of the literature on this clinically relevant topic.

In light of the high prevalence rate of falls in this patient population, the clinical implications indicate that falls prevention should be addressed both before and after surgery. Quality indicators for rehabilitation of TJA recommend balance and fall risk assessments before surgery with balance retraining during the recovery phase for both THA and TKA [51]. Relative to fall prevention programs in older adults in the community, few falls prevention programs specific to TJA exist. The majority of these TJA-specific programs have dealt with in-hospital falls and centred on the educational component [52–54]. Joint range, fear of falling and depression are modifiable risk factors for falls that could be specifically targeted. Although the etiology of falling is typically multifactorial, effective fall prevention interventions can reduce the number of falls by almost a third [55] and improve balance confidence in older adults [56]. However, the most effective time to deliver fall prevention programs during pre- and post-operative periods need to be identified.

## Conclusions

Substantial evidence for fall prevention exists for older adults; however, relatively few studies have reported prevalence of falls and risk factors in patients with TJA. The findings from this scoping review suggest there are a number of intrinsic risk factors for falling in patients with TJA, both pre- and post-operative, yet further investigation needs to examine extrinsic risk factors. A number of modifiable intrinsic risk factors were identified including fear of falling which should be targeted in fall prevention programs for TJA. The findings not only advocate for high quality large scale investigations examining the burden of falls, but also warrant research of interventions to prevent falls in this patient population.

## Supplementary information

**Additional file 1.** SearchStrategy_SUBMITTED.docx. Search strategies. Detailed search strategy for scoping review.

**Additional file 2.** Table S1_SUBMITTED.pdf. Table S1. Characteristics of included studies. Extracted data from included studies.

## Data Availability

The datasets used during the current review are available from the corresponding author on reasonable request.

## Abbreviations

CI: Confidence interval
FES-I: Falls Efficacy Scale International
OA: Osteoarthritis
OR: Odds ratio
ROM: Range of motion
THA: Total hip arthroplasty
TJA: Total joint arthroplasty
TKA: Total knee arthroplasty
WOMAC: Western Ontario and McMaster Universities Osteoarthritis Index

## References

[1] Carroll NV, Slattum PW, Cox FM (2005). The cost of falls among the community-dwelling elderly. J Manag Care Pharm.

[2] Muir SW, Berg K, Chesworth B, Klar N, Speechley M (2010). Quantifying the magnitude of risk for balance impairment on falls in community-dwelling older adults: a systematic review and meta-analysis. J Clin Epidemiol.

[3] Jones CA, Pohar S (2012). Health-related quality of life after total joint arthroplasty: a scoping review. Clin Geriatr Med.

[4] Moutzouri M, Gleeson N, Billis E, Tsepis E, Panoutsopoulou I, Gliatis J (2017). The effect of total knee arthroplasty on patients' balance and incidence of falls: a systematic review. Knee Surg Sports Traumatol Arthrosc.

[5] di Laura FG, Filardo G, Giunchi D, Fusco A, Zaffagnini S, Candrian C (2018). Risk of falls in patients with knee osteoarthritis undergoing total knee arthroplasty: a systematic review and best evidence synthesis. J Orthop.

[6] Guideline for the prevention of falls in older persons. American Geriatrics Society, British Geriatrics Society, and American Academy of Orthopaedic Surgeons Panel on Falls Prevention. J Am Geriatr Soc. 2001;49(5):664–72. 11380764

[7] Deandrea Silvia, Lucenteforte Ersilia, Bravi Francesca, Foschi Roberto, La Vecchia Carlo, Negri Eva (2010). Risk Factors for Falls in Community-dwelling Older People. Epidemiology.

[8] de Zwart AH, van der Esch M, Pijnappels MA, Hoozemans MJ, van der Leeden M, Roorda LD, Dekker J, Lems WF, van Dieen JH (2015). Falls associated with muscle strength in patients with knee osteoarthritis and self-reported knee instability. J Rheumatol.

[9] Leveille SG, Jones RN, Kiely DK, Hausdorff JM, Shmerling RH, Guralnik JM, Kiel DP, Lipsitz LA, Bean JF (2009). Chronic musculoskeletal pain and the occurrence of falls in an older population. JAMA.

[10] Picorelli AMA, Hatton AL, Gane EM, Smith MD (2018). Balance performance in older adults with hip osteoarthritis: a systematic review. Gait Posture.

[11] Manlapaz DG, Sole G, Jayakaran P, Chapple CM (2019). Risk factors for falls in adults with knee osteoarthritis: a systematic review. PM R.

[12] Arnold CM, Gyurcsik NC (2012). Risk factors for falls in older adults with lower extremity arthritis: a conceptual framework of current knowledge and future directions. Physiother Can.

[13] Leveille SG, Bean J, Bandeen-Roche K, Jones R, Hochberg M, Guralnik JM (2002). Musculoskeletal pain and risk for falls in older disabled women living in the community. J Am Geriatr Soc.

[14] Chu L-W, Chi I, Chiu AY (2005). Incidence and predictors of falls in the Chinese elderly. Ann Acad Med Singap.

[15] Tinetti M. E., Richman D., Powell L. (1990). Falls Efficacy as a Measure of Fear of Falling. Journal of Gerontology.

[16] Scheffer AC, Schuurmans MJ, van Dijk N, van der Hooft T, de Rooij SE (2008). Fear of falling: measurement strategy, prevalence, risk factors and consequences among older persons. Age Ageing.

[17] Legters K (2002). Fear of falling. Phys Ther.

[18] Levinger P, Menz HB, Wee E, Feller JA, Bartlett JR, Bergman NR (2011). Physiological risk factors for falls in people with knee osteoarthritis before and early after knee replacement surgery. Knee Surg Sports Traumatol Arthrosc.

[19] Lo CWT, Tsang WWN, Yan CH, Lord SR, Hill KD, Wong AYL (2019). Risk factors for falls in patients with total hip arthroplasty and total knee arthroplasty: a systematic review and meta-analysis. Osteoarthr Cartil.

[20] Arksey H, O'Malley L (2005). Scoping studies: towards a methodological framework. Int J Soc Res Methodol.

[21] Morrison A, Polisena J, Husereau D, Moulton K, Clark M, Fiander M, Mierzwinski-Urban M, Clifford T, Hutton B, Rabb D (2012). The effect of English-language restriction on systematic review-based meta-analyses: a systematic review of empirical studies. Int J Technol Assess Health Care.

[22] Covidence systematic review software, Veritas Health Innovation, Melbourne, Australia. Available at www.covidence.org.

[23] McHugh ML (2012). Interrater reliability: the kappa statistic. Biochemia Medica.

[24] Scottish Intercollegiate Guidelines Network (SIGN). Guidelines Checklist 3: Cohort Studies; Edinburgh: Scottish Intercollegiate Guidelines Network; 2014. Available at https://www.sign.ac.uk/checklists-and-notes.html.

[25] Ikutomo H, Nagai K, Nakagawa N, Masuhara K (2015). Falls in patients after total hip arthroplasty in Japan. J Orthop Sci.

[26] Soison A, Riratanapong S, Chouwajaroen N, Chantowart C, Buranapiyawong L, Kaewkot S, Kosuwon W (2014). Prevalence of fall in patients with total knee arthroplasty living in the community. J Med Assoc Thail.

[27] Pozzi F, Abujaber S, Fenstermacher S, Zeni J (2015). Relationship between functional performance and falls in female patients with end stage hip osteoarthritis. Osteoarthr Cartil.

[28] Mitchell S, McCaskie A, Francis R, Peaston R, Birrell F, Lingard E (2007). The need for a falls prevention programme for patients undergoing hip and knee replacement surgery. J Orthop Nurs.

[29] Hill KD, Wee E, Margelis S, Menz HB, Bartlett J, Bergman NR, McMahon S, Hare DL, Levinger P (2016). Falls in people prior to undergoing total hip or total knee replacement surgery: frequency and associated factors. Journal of Clinical Gerontology and Geriatrics.

[30] Tsonga T, Michalopoulou M, Malliou P, Godolias G, Kapetanakis S, Gkasdaris G, Soucacos P (2015). Analyzing the history of falls in patients with severe knee osteoarthritis. Clinics in Orthopedic Surgery.

[31] Matsumoto H, Okuno M, Nakamura T, Yamamoto K, Hagino H (2012). Fall incidence and risk factors in patients after total knee arthroplasty. Arch Orthop Trauma Surg.

[32] Tsonga T, Michalopoulou M, Kapetanakis S, Giovannopoulou E, Malliou P, Godolias G, Soucacos P (2016). Reduction of falls and factors affecting falls a year after total knee arthroplasty in elderly patients with severe knee osteoarthritis. Open Orthop J.

[33] Swinkels A, Newman JH, Allain TJ (2008). A prospective observational study of falling before and after knee replacement surgery. Age Ageing.

[34] Pearson M, Latham SK, Smith TO (2016). Are people following hip and knee arthroplasty at greater risk of experiencing a fall and fracture? Data from the osteoarthritis initiative. Arch Orthop Trauma Surg.

[35] Riddle D, Golladay G (2016). A longitudinal comparative study of falls in persons with knee arthroplasty and persons with or at high risk for knee osteoarthritis. Age Ageing.

[36] Powell LE, Myers AM (1995). The Activities-specific Balance Confidence (ABC) Scale. The J Gerontol Ser A, Biol Sci Med Sci.

[37] Yardley L, Beyer N, Hauer K, Kempen G, Piot-Ziegler C, Todd C (2005). Development and initial validation of the falls efficacy scale-international (FES-I). Age Ageing.

[38] Hill KD, Schwarz JA, Kalogeropoulos AJ, Gibson SJ (1996). Fear of falling revisited. Arch Phys Med Rehabil.

[39] Tinetti ME, Speechley M, Ginter SF (1988). Risk factors for falls among elderly persons living in the community. N Engl J Med.

[40] Delbaere K, Close JCT, Heim J, Sachdev PS, Brodaty H, Slavin MJ, Kochan NA, Lord SR (2010). A multifactorial approach to understanding fall risk in older people. J Am Geriatr Soc.

[41] Memtsoudis SG, Dy CJ, Ma Y, Chiu Y-L, Della Valle AG, Mazumdar M (2012). In-hospital patient falls after total joint arthroplasty: incidence, demographics, and risk factors in the United States. The J Arthroplasty.

[42] Mandl LA, Lyman S, Quinlan P, Bailey T, Katz J, Magid SK (2013). Falls among patients who had elective orthopaedic surgery: a decade of experience from a musculoskeletal specialty hospital. J Orthop Sports Phys Ther.

[43] Muraki S, Akune T, Oka H, En-yo Y, Yoshida M, Nakamura K, Kawaguchi H, Yoshimura N (2011). Prevalence of falls and the association with knee osteoarthritis and lumbar spondylosis as well as knee and lower back pain in Japanese men and women. Arthritis Care & Research.

[44] Patel KV, Phelan EA, Leveille SG, Lamb SE, Missikpode C, Wallace RB, Guralnik JM, Turk DC (2014). High prevalence of falls, fear of falling, and impaired balance in older adults with pain in the United States: findings from the 2011 National Health and aging trends study. J Am Geriatr Soc.

[45] Chiacchiero M, Dresely B, Silva U, DeLosReyes R, Vorik B (2010). The relationship between range of movement, flexibility, and balance in the elderly. Topics Geriatr Rehabil.

[46] Zijlstra GR, van Haastregt JCM, van Eijk JTM, van Rossum E, Stalenhoef PA, GIJM K (2007). Prevalence and correlates of fear of falling, and associated avoidance of activity in the general population of community-living older people. Age Ageing.

[47] Brouwer BJ, Walker C, Rydahl SJ, Culham EG (2003). Reducing fear of falling in seniors through education and activity programs: a randomized trial. J Am Geriatr Soc.

[48] Kumar A, Delbaere K, Zijlstra GA, Carpenter H, Iliffe S, Masud T, Skelton D, Morris R, Kendrick D (2016). Exercise for reducing fear of falling in older people living in the community: Cochrane systematic review and meta-analysis. Age Ageing.

[49] Hale WA, Delaney MJ, Cable T (1993). Accuracy of patient recall and chart documentation of falls. The Journal of the American Board of Family Practice.

[50] Ganz DA, Higashi T, Rubenstein LZ (2005). Monitoring falls in cohort studies of community-dwelling older people: effect of the recall interval. J Am Geriatr Soc.

[51] Westby MD, Marshall DA, Jones CA (2018). Development of quality indicators for hip and knee arthroplasty rehabilitation. Osteoarthr Cartil.

[52] Johnson RL, Duncan CM, Ahn KS, Schroeder DR, Horlocker TT, Kopp SL (2014). Fall-prevention strategies and patient characteristics that impact fall rates after total knee arthroplasty. Anesth Analg.

[53] Clarke HD, Timm VL, Goldberg BR, Hattrup SJ (2012). Preoperative patient education reduces in-hospital falls after total knee arthroplasty. Clin Orthop Relat Res.

[54] Kim TE, Mariano ER (2014). Developing a multidisciplinary fall reduction program for lower-extremity joint arthroplasty patients. Anesthesiol Clin.

[55] Campbell AJ, Robertson MC (2007). Rethinking individual and community fall prevention strategies: a meta-regression comparing single and multifactorial interventions. Age Ageing.

[56] Bula CJ, Monod S, Hoskovec C, Rochat S (2011). Interventions aiming at balance confidence improvement in older adults: an updated review. Gerontology.

