# Supplementary material for: Falls and fear of falling in older adults with total joint arthroplasty: a scoping review
Source: BMC Musculoskelet Disord. 2019 Dec 12;20:599. doi: 10.1186/s12891-019-2954-9 (PMC6909481; doi:10.1186/s12891-019-2954-9)
Supplement: Supplementary file 1 — Additional file 1. SearchStrategy_SUBMITTED.docx. Search strategies. Detailed search strategy for scoping review. [file 12891_2019_2954_MOESM1_ESM.docx]

Additional file 1

**APPENDIX A:  Search strategies**

**Pubmed** (for epub ahead of print only)

Date searched: April 6, 2017

Results: 5

pubstatusaheadofprint AND osteoarthritis AND (hip arthroplasty or joint arthroplasty or knee arthroplasty or hip replacement or knee replacement or joint replacement) AND (fall OR falls OR falling OR fallers OR faller)

**Medline** (Epub Ahead of Print, In-Process & Other Non-Indexed Citations, Ovid MEDLINE(R) Daily and Ovid MEDLINE(R) 1946 to Present)

Date searched: April 6, 2017

Results: 174

1. exp Osteoarthritis/

2. osteoarthriti*.mp.

3. 1 or 2

4. hip joint/ or hip/

5. Knee Joint/ or Knee/

6. "prostheses and implants"/ or joint prosthesis/

7. arthroplasty/ or arthroplasty, replacement/

8. (4 or 5) and (6 or 7)

9. hip prosthesis/ or knee prosthesis/

10. arthroplasty, replacement, hip/ or arthroplasty, replacement, knee/

11. ((total or complete) adj6 (hip or hips or knee or knees) adj6 (arthroplast* or prosthe* or replace* or implant*)).mp.

12. (((total or complete) adj6 joint adj6 (arthroplast* or prosthe* or replace* or implant*)) and (hip or hips or knee or knees)).mp.

13. (TKA or THA or TJA or TKR or TJR or THR).ti.

14. or/8-13

15. Accidental Falls/

16. (fall* or activities-specific balance confidence).mp.

17. 15 or 16

18. 3 and 14 and 17

**CENTRAL** (OVID Platform) EBM Reviews - Cochrane Central Register of Controlled Trials May 2016

Date searched: inception to July 5, 2016

Results: 25

Search strategy identical to Medline above

**Cochrane Central Register of Controlled Trials (Wiley Interface)**

Date searched: July 2016 - April 2017

Results: 2

osteoarthrit*:ti,ab,kw

and

fall* or "activities-specific balance confidence":ti,ab,kw

and

"hip arthroplast*" or "joint arthroplast*" or "knee arthroplast*" or "hip replacement*" or "knee replacement*" or "joint replacement*":ti,ab,kw Publication Year from 2016 to 2017, in Trials

**Embase**  (OVID Platform) 1974 to 2017 April 05

Date searched: April 6, 2017

Results: 293

1. exp osteoarthritis/

2. osteoarthriti*.mp.

3. 1 or 2

4. exp knee arthroplasty/ or exp total hip prosthesis/ or exp total knee replacement/

5. exp knee arthroplasty/ or exp hip arthroplasty/ or exp total hip prosthesis/ or exp total knee replacement/

6. knee prosthesis/ or hip prosthesis/

7. (arthroplasty/ or joint prosthesis/) and (knee/ or hip/)

8. ((total or complete) adj6 (hip or hips or knee or knees) adj6 (arthroplast* or prosthe* or replace* or implant*)).mp.

9. (((total or complete) adj6 joint adj6 (arthroplast* or prosthe* or replace* or implant*)) and (hip or hips or knee or knees)).mp.

10. (TKA or THA or TJA or TKR or TJR or THR).ti.

11. or/4-10

12. falling/

13. fall risk assessment/

14. (fall* or activities-specific balance confidence).mp.

15. or/12-14

16. 3 and 11 and 15

**CINAHL Plus with Full Text**  (EBSCO Interface)

Searched: April 6, 2017

Results: 45

*Search modes: Boolean/Phrase*

S1 (MH "Osteoarthritis+") OR osteoarthrit*

S2 ( (MH "Arthroplasty, Replacement, Hip") OR (MH "Arthroplasty, Replacement, Knee") ) OR ( (total or complete) n6 (hip or hips or knee or knees) n6 (arthroplast* or prosthe* or replace* or implant*) ) OR ( ((total or complete) n6 joint n6 (arthroplast* or prosthe* or replace* or implant*)) and (hip or hips or knee or knees) ) OR ( TKA or THA or TJA or TKR or TJR or THR )

S3. ( (MH "Accidental Falls") OR (MH "Safety Status: Falls Occurrence (Iowa NOC)") OR (MH "Fall Risk (Saba CCC)") OR (MH "Fall Risk Assessment Tool") OR (MH "Hendrich Fall Risk Model") OR (MH "Morse Fall Scale") ) OR ( fall* or activities-specific balance confidence )

S4. S1 AND S2 AND S3

**Web of Science** (ISI Interface)

Searched: April 6, 2017

Databases: Web of Science Core Collection

Results: 82

#1 TI=(TKA OR THA OR TJA OR TKR OR TJR OR THR)

#2 TS=((total or complete) NEAR/4 joint NEAR/4 (arthroplast* or prosthe* or replace* or implant*)) AND TS=(hip or hips or knee or knees)

#3 TS=((total or complete) NEAR/4 (hip or hips or knee or knees) NEAR/4 (arthroplast* or prosthe* or replace* or implant*))

#4 TS=(TKA or THA or TJA or TKR or TJR OR THR) AND TS=((joint or hip or hips or knee or knees) NEAR/4 (arthroplast* or prosthe* or replace* or implant*))

#5 #1 OR #2 OR #3 OR #4

#6 TS=osteoarthrit*

#7 TS=(fall* or "activities-specific balance confidence")

#8 #5 AND #6 AND #7

**SCOPUS**

Searched: April 6, 2017

Results: 240

TITLE-ABS-KEY ( osteoarthrit* ) AND ( TITLE-ABS-KEY ( fall* OR "activities-specific balance confidence" ) AND TITLE-ABS-KEY ( "hip arthroplast*" OR "joint arthroplast*" OR "knee arthroplast*" OR "hip replacement*" OR "knee replacement*" OR "joint replacement*" ) OR TITLE ( tja OR tka OR tha OR tkr OR tjr OR thr ) )

Total: 866
