# Supplementary material for: Falls and fear of falling in older adults with total joint arthroplasty: a scoping review
Source: BMC Musculoskelet Disord. 2019 Dec 12;20:599. doi: 10.1186/s12891-019-2954-9 (PMC6909481; doi:10.1186/s12891-019-2954-9)
Supplement: Supplementary file 2 — Additional file 2. Table S1_SUBMITTED.pdf. Table S1. Characteristics of included studies. Extracted data from included studies. [file 12891_2019_2954_MOESM2_ESM.pdf]

Additional File 2

Table S1. Characteristics of included studies

Author	Study Design	Sample Characteristics	Joint replaced	Timeframe: Pre-operative or post-operative	Recall time for falls	Number of falls	Measures	Results			Quality of Study
**Pre-operative studies**											
Hill et al. (2016)<br><br>*Australia*	Cross sectional	n=282<br><br>Age: 67.3 ±8.6 yrs<br><br>Female n =155 (55%)	Hip (n=85)<br><br>Knee (n=197)<br><br>Total (n=282)	pre-operative = 2-4 weeks	12 months	116 (41%) had one fall or more in the past 12 months		***TKA Non-fallers***	***TKA Fallers***	***p-value***	
							Falls Efficacy Scale: International<br>7-28 (7: no concern)	11.4 ± 3.8	13.5 ± 5.6	0.005	
							Activities- Specific Balance Confidence Scale<br>0-100 (0: least fearful)	71.2 ± 22.1	63.8 ± 20.6	0.051	
							WOMAC Function, VAS Scale<br>0-1700 (0: no difficulty with function)	671.7 ± 280.0	807.4 ± 282.00	0.020*	
							Self-perceived quality of life (SF-36 Mental)<br>0-100 (0: lowest quality)	54.9 ± 9.7	50.6 ± 13.1	0.030*	
							Pain (Pain Catastrophizing Scale)<br>0-52 (0:least catastrophizing)	11.1 ± 9.5	14.4 ± 11.5	0.021**	Acceptable
							Cardiac Depression Scale (CDS)<br>26 – 182 (26: least depressive mood)	S68.2 ± 21.2	79.5 ± 23.9	0.007*	
							** One-way ANOVA post hoc: significant difference between multiple fallers and other groups*<br>*** One-way ANOVA post hoc: significant difference between all three groups*<br><br>**Not significant:** TKA patients- WOMAC pain/ stiffness/ total; SF-36 Physical Component Score; Tampa Scale for Kinesiophobia; IPEQ Incidental Activity; IPEQ planned activity / total activity were not significantly different between non-fallers and multiple fallers.<br><br>**Not significant:** THA patients - WOMAC pain/ stiffness/ function/ total; Pain Catastrophizing Scale; SF-36 Physical Component score/ Mental Component Score; Tampa Scale for Kinesiophobia; Cardiac Depression Scale; IPEQ incidental/ planned/ total				

<table>
<tr>
<td>Mitchell et al.
(2007)

United Kingdom</td>
<td>Cross Sectional</td>
<td>n = 199

Age: 72 (95% CI: 71.6, 72.7) yrs

Female 111 (55.6%)</td>
<td>Hip (n=84)

Knee (n=115)</td>
<td>pre-operative time points not specified</td>
<td>4 weeks</td>
<td>75 (39%) of pre-op patients fell at least once</td>
<td>
<table>
<tr><th></th><th>Non fallers</th><th>Fallers</th><th>p-value</th></tr>
<tr><td>WOMAC Pain*
0 – 100, 0 least pain</td><td>45</td><td>33</td><td>0.0012</td></tr>
<tr><td>WOMAC Function*
0 – 100, 0 no difficulty with function</td><td>41</td><td>33</td><td>0.0001</td></tr>
<tr><td>Self-perceived quality of life -36 *</td><td>28</td><td>20</td><td><0.05</td></tr>
<tr><td>Physical Functioning</td><td>33</td><td>25</td><td><0.05</td></tr>
<tr><td>Bodily Pain</td><td>17</td><td>7</td><td><0.05</td></tr>
<tr><td>Role Physical</td><td>60</td><td>52</td><td><0.05</td></tr>
<tr><td>General Health</td><td>48</td><td>40</td><td><0.05</td></tr>
<tr><td>Vitality</td><td>56</td><td>36</td><td><0.05</td></tr>
<tr><td>Role Emotional</td><td></td><td></td><td></td></tr>
<tr><td>Timed Up and Go – mean (95% CI)</td><td>16 (14.9, 17.0)</td><td>19.8 (16.8, 22.8)</td><td>0.02</td></tr>
<tr><td>Hypnotic/Antidepressant Medication – n (95% CI)</td><td>8/119 (7)</td><td>13/75 (17)</td><td>0.02</td></tr>
<tr><td>Number of Comorbid Conditions – mean (95% CI)</td><td>1.9 (1.6, 2.1)</td><td>2.6 (2.2, 3.0)</td><td>0.0025</td></tr>
<tr><td>Self-Reported Depression – n (95% CI)</td><td>14/115 (12)</td><td>17/69 (25)</td><td>0.0025</td></tr>
<tr><td>Number of Geriatric Problems – mean (95% CI)</td><td>1.2 (0.9, 1.4)</td><td>1.8 (1.5, 2.1)</td><td>0.0016</td></tr>
<tr><td>Self-Reported Memory Problem – n (95% CI)</td><td>11/114 (10)</td><td>17/72 (24)</td><td>0.010</td></tr>
<tr><td>Self-Reported Balance Problem – n (95% CI)</td><td>37/114 (33)</td><td>42/72 (58)</td><td>0.0005</td></tr>
</table>

Not significant: WOMAC stiffness, SF-Social Functioning, SF-Role Emotional (p>0.05), age, sex, joint being replaced, osteoporosis at any site, use of parathyroid hormone, use of vitamin D, BMI, and number of prescription medications were not statistically different between non-fallers and fallers.
<table>
<tr><th></th><th>Adjusted OR (for sex and gender) for factors associated with falling</th><th>p-value</th></tr>
<tr><td>Number of comorbid conditions > 2</td><td>2.2 (1.03, 4.8)</td><td><0.05</td></tr>
<tr><td>Self-reported balance problem</td><td>2.5 (1.2, 5.1)</td><td><0.05</td></tr>
</table>

Not significant: Self reported depression, Hypnotic/antidepressant medication, more than one geriatric problem, and self-reported memory problem were factors that were not significantly associated with falling.
</td>
<td>Poor</td>
</tr>
<tr>
<td>Pozzi et al
(2015)
USA</td>
<td>Cross Sectional</td>
<td>n=31

Age: 65 ± 8 yrs

Female 31 (100%)</td>
<td>Hip (n=31)</td>
<td>Pre-operative = 2 weeks</td>
<td>6 months</td>
<td>7 (22.5%) of pre-op patients reported at least one fall within 6 months of survey</td>
<td>
<table>
<tr><th></th><th>Non-fallers (n=24)</th><th>Fallers (n=7)</th><th>t-test</th><th>Correlation with fall (r, p-value)</th></tr>
<tr><td>Age</td><td>63 ± 8</td><td>71 ± 6</td><td>0.01</td><td>0.40, 0.03</td></tr>
<tr><td>Timed up and go (s)</td><td>10.9 ± 3.43</td><td>17.63 ± 11.13</td><td>>0.01</td><td>0.45, 0.01</td></tr>
<tr><td>Stair climbing (s)</td><td>22.64 ± 9.07</td><td>41.4 ± 20.4</td><td>>0.01</td><td>0.54, >0.01</td></tr>
<tr><td>6 minute walk test (m)</td><td>399.16 ±86.19</td><td>264.36 ±145.85</td><td>>0.01</td><td>-0.5, >0.01</td></tr>
</table>
</td>
<td>Poor</td>
</tr>
</table>

							Total hip ROM, degrees — 156.08 ±26.94 — 135.85 ±13.45 — 0.03 — -0.33, 0.06<br>Knee extension on surgical side of hip, Nm/Kg — 2.15 ± 1.13 — 1.33 ± 0.63 — 0.04 — -0.32, 0.08<br>Knee extension on non-surgical side of hip, Nm/Kg — 1.04 ± 2.19 — 2.08 ± 1.24 — 0.04 — -0.31, 0.08<br><br>**Not significant**: BMI; Hip Harris Score; Hip Outcome Score; low back pain on a 1-10 scale; hip pain on 1-10 scale for both surgical and non-surgical side; knee pain on 1-10 scale on surgical and non-surgical side; knee abductor strength on surgical and non-surgical side; are all not significantly different between non-fallers and fallers.	
Tsonga et al (2015)<br><br>*Greece*	Cross Sectional	n=68<br><br>Age: 73 ±5.8yrs<br><br>Female 57 (83.8%)	Knee (n=68)	Pre-operative = 1 month	12 months	43 (63.2%) had one or more falls in the past 12 months	**Mean ± SD**<br>SF-36 Physical — 34.5 ± 6.99<br>SF-36 Mental — 36.36 ± 9.83<br>WOMAC (VAS Scale)<br>Total — 822.79 ± 317.31<br>Pain — 226.10 ± 85.24<br>Stiffness — 56.62 ± 43.54<br>Physical function — 540.07 ± 255.18<br>Timed Up and Go Performance Test (seconds) — 13.05 ± 4.13<br><br>**Not significant**: Gender; age; BMI; presence of pain elsewhere in the body; number chronic diseases; having a social environment; previous arthroplasty; SF-36 physical & mental component scores; WOMAC pain/ stiffness/ physical function; Timed Up and Go were not significantly different between non-fallers and fallers	Acceptable
**Pre and post-operative studies**								
Levinger et al. (2011)<br><br>*Australia*	Prospective Cohort	n=62<br><br>Age: 66 ±7 yrs<br><br>Female n =30 (48%)	Knee (n=35)<br><br>Non-Surgical Controls (n=27)	pre-operative time not specified<br><br>post-operative = 4 months	12 months	17 (48%) of pre-op TKA patients fell<br><br>8 (30%) of non-surgical controls fell<br><br>*Timeframe not reported for pre-op patients<br>**Post-op prevalence of falls not measured	**Pre-operative** — **Post-operative** — **Non-surgical control**<br>FES-I 7-28 (7: no concern) — 11.4 ± 3.0 *+ — 9.7 ± 2.9* — 7.6 ± 1.2<br>WOMAC Pain - VAS (range 0-500, 500 worst) — 192.5 ± 106.0 + — 171.5 ± 278.2 — -<br>WOMAC Stiffness - VAS (range 0-200, 200 worst) — 95.4 ± 46.7 + — 48.6 ± 37.3 — -<br>WOMAC Function - VAS (0-1,700) — 609.0 ± 325.9 + — 278.6 ± 236.0 — -<br>WOMAC Total - VAS (0-2,400) — 896.9 ± 430.4 + — 498.7 ±498.5 — -<br>Assessment of Quality of Life — 0.8 ± 0.0 + — 0.7 ± 0.1* — 0.8 ± 0.1<br>Incidental and Planned Activity Questionnaire: Incidental — 38.5 ± 19.8*+ — 8.3 ± 14.5* — 19.5 ± 13.9<br>Incidental and Planned Activity Questionnaire: Total — 44.3 ± 20.6 *+ — 12.1 ± 16.1 — 25.9 ± 16.3<br><br>***Not significant*** **:** IPAQ planned was not significantly different between control and post-operative group.<br>** Significantly different to control*<br>*+Significantly different to post-surgery*	Acceptable

Riddle et al. (2016)<br><br>*USA*	Retrospective Cohort	Cases: n=413<br><br>Age: 63.9 ±6.8 yrs<br><br>Female: 251 (60.7%)<br><br>Controls: n=4,200<br><br>Age: 60.8 ±9.2 yrs<br><br>Female: 2,446 (58.2%)<br><br>Controls were OA patients and individuals at risk of OA (54% had no OA and 49.1% had grade 2 or more) that did not undergo TKA during the study period	Knee arthroplasty patients (n=413)	pre-operative = 4 years<br>post-operative = 4 years	12 months	8-23% for men, and 15-23% for women fell at least once in the past year	***Non-TKA (OR, 95% CI)*** / ***TKA (OR, 95% CI)***<br>*1 Fall (reference group: no falls)*<br>Age: 1.00, (0.99,1.01) / 1.02 (1.01, 1.03)*<br>Male Sex: 0.60 (0.49, 0.74)* / 0.83 (0.67, 1.02)<br>No Narcotic Use: 0.63 (0.41, 0.97)* / 0.41 (0.33, 0.50)<br>No Prior Falls: 0.74 (0.47, 1.15)* / 0.78 (0.55, 1.20)*<br><br>***Not significant*** *(OR, 95% CI)*: PASE score; depressive symptoms; repeated chair stand; no comorbidity; no knee replacement / PASE score; depressive symptoms; repeated chair stand; no comorbidity; no knee replacement<br><br>*2+ Falls (reference group: no falls)*<br>Age: 0.98 (0.97, 0.99)* / 0.99 (0.98, 1.01)*<br>Depressive symptoms: 1.05 (1.04, 1.07)* / 1.04 (1.03, 1.05)*<br>No comorbidity: 0.77 (0.60, 0.97)* / 0.80 (0.66, 1.08)<br>No narcotic use: 0.53 (0.34, 0.82)* / 0.89 (0.55, 1.44)<br>No prior falls: 0.16 (0.13, 0.21)* / 0.14 (0.10, 0.18)*<br><br>***Not significant*** *(OR, 95% CI)*: Male sex; PASE score; repeated chair stand; no knee replacement / Male sex; PASE score; repeated chair stand; no knee replacement	Acceptable

Swinkles et al. (2009)<br><br>*UK*	Prospective Cohort	n=99<br><br>Age: 73.4 ± 4.9 yrs<br><br>Female: 63(64%)	Knee (n=99)	pre-operative = 3 months<br><br>post-operative =12 months	1 month	Pre-op: 24 (24%) fell<br><br>Post-op: 11 (13%) fell	***3 Months Pre-Op*** / ***3 Months Post-Op*** / ***6 Months Post-Op*** / ***12 Months Post-Op***<br>ABC-UK (0-100, 100 Best)<br>Fallers: 53.1±26.4 / 64.6±18.7 / 66.0±23.2 / 61.2±30.4<br>Non-Fallers: 64.4±24.3 / 67.6±22.8 / 71.2±25.8 / 76.7±22.7<br><br>***Pre- Operative Fallers** (n=24)* / ***Pre-Operative Non-Fallers** (n=75)*<br>ABC – UK (0-100, 100 best): 53.1 ± 26.4 / 64.4 ±24.3<br>Geriatric Depression Scale (0-15, 15 worst): 5.4 ± 2.6 / 3.4 ± 2.5<br>WOMAC Pain (Likert) (0-20, 20 worst): 11.7 ± 2.7 / 10.4 ± 3.3<br>WOMAC Stiffness (Likert) (0-8, 8 worst): 5.4 ± 1.2 / 4.8 ± 1.6<br>WOMAC Function (Likert) (0-68, 68 worst): 42.7 ± 9.4 / 35.9 ± 11.4<br>WOMAC Total (Likert) (0-96, 96 worst): 59.5 ± 11.8 / 51.0 ± 15.5<br>Patients reporting falling 4 months prior to entry onto waitlist for TKA: 12/23 / 4/75<br><br>***Not significant:****(adjusted OR and 95% CI for pre-operative and post-operative patients):* female gender; age; number of comorbid conditions; number of baseline medications including cardiovascular medications, anti-depressants; self-reported eye problems; self-reported hearing problems.	High	
Tsonga et al (2016)<br><br>*Greece*	Prospective Cohort	n=68<br><br>Age: 73 ± 5.28 yrs<br><br>Female: 57 (83.8%)	Knee (n=68)	pre-operative = 2 weeks<br><br>post-operative = 1-12 months	1 month	Pre-op: 43 (63%) fell<br><br>Post-op: 15 (22%) fell	***Pre-operative*** / ***1 year Post-operative***<br>Fear of Falling (Yes): 56 (82.4%) / 30 44.1%<br>Fear of Falling –Activities Specific Balance Confidence Scale (0-100, 0 least confidence), mean ± SD: 63.76 ± 20.14 / 81.49 ± 16.24<br>*Fallers at 1 year post-op* / *Non-Fallers at 1 year post-op*<br>Fear of Falling –Activities Specific Balance Confidence: 79.50 ± 13.99 / 82.05 ± 16.90<br>Fearful of Falling (Yes/No): 11 (73.3%) / 19 (35.8%)<br>History of Falls (Yes/No): 12 (30.2%) / 30 (69.8%)<br>**Not significant:** Age; gender; BMI; pain elsewhere in the body; chronic diseases ; complications during surgery; social environment; presence of social environment; previous arthroplasty surgery; SF-36 Physical & Mental component scores; WOMAC pain/ stiffness/ physical function; PASE physical activity questionnaire; Timed Up and Go, and Berg Balance scale were not significantly different between fallers and non-fallers at 1 year post-op.	Acceptable	< < 0<br>0<br><

							O.R, 95%CI (multiple logistic regression of falling status at 1 year post-op) stepwise method<br>Age: 1.13, (0.98, 1.30)<br>Fear of Falling: 11.90 (2.20, 64.20<br>Falling Status: *7.23 (1.28, 41.01)*	
**Post-operative studies**								
Ikutomo et al (2015)<br><br>*Japan*	Cross Sectional	n=214<br><br>Age: 66 ±8.7 yrs<br><br>Female 203 (95%)	Hip (n=214)	post-operative= 1 year	12 months	77 (36%) of post-op patients fell	| | ***Non-Fallers*** | ***Fallers*** | ***p-value*** | Age (yrs) | 86.3 ± 8.2 | 64.7 ± 8.7 | 0.004<br>Height (cm) | 153 ± 6.2 | 154.8 ± 5.9 | 0.037<br>Medication, n (%) | 63 (81.8) | 69 (50.4) | <0.001<br>Less than one comorbid medical condition, n (%) | 30 (39) | 73 (53.3) | 0.044<br>Post-operative duration, years (interquartile range) | 4 (2-7) | 5 (2-8) | 0.023<br>Total Oxford Hip Score | 16 (14-19) | 14 (12-17) | <0.001<br>Use of a walking aid, n (%) | 27 (35.1) | 25 (18.2) | 0.006<br>Walking capacity –"can you walk for more than 60 minutes without stopping?" n (%) | 32 (41.6) | 88 (64.2) | 0.001<br><br>**Not significant*:*** Sex; body weight; BMI; THA side were not different between non-fallers and fallers.<br><br>| ***Adjusted OR (95%CI)*** *(for sex and gender) for factors associated with falling* | *p*-value<br>Medications | 4.09 (1.90, 8.80) | <0.001<br>Post Operative Duration, years (IQR) | 0.89 (0.81, 0.98) | 0.014	Poor
Matsumoto (2012)<br><br>*Japan*	Prospective Cohort	n=74<br><br>Age: 75 ± 6 yrs<br><br>Female: 57 (88%)	Knee (n=70)	post-operative =6-11 months	1 month, for 6 months	23 (32.9%) fell once or more during a 6-month observation period	| ***Fallers*** | ***Non-Fallers*** | ***p-value***<br>Modified Falls Efficacy Scale (M-FES) | 122.2 ± 27.1 | 123.3 ± 23.3 | 0.874<br>Post operative flexion | 10.2 ± 16.1 | 119.5 ± 14.1 | 0.016<br>Post operative flexion and extension | 100.6 ± 18.4 | 109.7 ± 15.9 | 0.037<br>ROM of the ankle (°) – plantar flexion | 55.2 ± 6.1 | 59.1 ± 6.1 | 0.014<br>**Not significant*:*** Age; gender; BMI; TKA side; diagnosis of OA or RA; mean time since surgery; prior hip surgery; total number of prescribed medications; hearing problems; eye problems; cardiac disease; diabetes; hypertension; ambulation; pre-operative knee flexion; pre-operative knee extension; pre-operative range of flexion and extension; post operative extension; ankle dorsiflexion; joint instability; knee extensor strength; hallux valgus; limited ankle mobility; kyphosis; one-leg stand; 10 minute walking speed; step length; JKOM total score; pain; limitation of activity; restriction of participation; and Geriatric Depression Scale; were not statistically different between fallers and non-fallers.	High

							OR (95% CI) *p-value*<br>Knee flexion (post op) (10 degree groups, 80-140) 0.277 (0.088-0.869) 0.028<br>Knee flexion and extension (post op (10 degree groups, 60-135) 2.308 (0.847-6.289) 0.102<br>Ankle plantar flexion (5 degree groups, 40-70) 0.594 (0.374-0.945) 0.028	
Soison et al (2014)<br><br>*Thailand*	Cross Sectional	n=54<br><br>Age: 67 ±8 yrs<br><br>Female 46 (85%)	Knee (n=54)	post–operative= 7-73 months	12 months	23 (42%) of post-op patients fell	*Non-Fallers* *Fallers* *p-value*<br>Height (cm) 156.06 ± 7.29 154.26 ± 4.22 <0.005<br>Limited Knee Joint Motion (case) 2 7 <0.05<br>**Not significant**: Age, weight, BMI, time after surgery, VAS, WOMAC pain, stiffness, function, total, satisfaction score (maximum score 10), vision problems<br><br>*Coefficient of association between risk factor and falls* *Standard Error* *p value*<br>WOMAC Pain 0.048 0.021 <0.05<br>WOMAC Stiffness 0.141 0.049 <0.025<br>Limit Knee Joint Motion (flexion <120° and/or joint was not able to fully extend 0°) 0.594 0.227 <0.025<br><br>**Not significant:** Sex; vision problems; BMI; VAS; WOMAC function/ total score.	Poor
Smith et al (2014)<br><br>*United Kingdom*	Retrospective Cohort	n=269<br><br>Age: 67 ± 9 yrs<br><br>Female 112 (41.6%)	Hip (n=104)<br><br>Knee (n=165)	post-operative = 12-72 months	12 months	26 (25%) of post-op THA patients fell at least once<br><br>43 (26.1%) of post-op TKA patients fell at least once	*Falls in THA patients* *Falls in TKA patients*<br>Bisphosphonate use (OR, 95% CI: *p* value) 1.28 (1.03, 1.58) 0.02 1.23 (1.03, 1.58) 0.03<br>Knee OA in the past (OR, 95% CI: *p* value) 1.51 (1.06, 2.04) 0.02 -<br>**Not significant:** Age; gender; marital status; employment; diagnosis of hip OA; race Age; gender; marital status; employment; previous hip arthroplasty; diagnosis of hip OA; diagnosis of knee OA; race	Acceptable
